# Novel players in the development of chemoresistance in ovarian cancer: ovarian cancer stem cells, non-coding RNA and nuclear receptors

**DOI:** 10.20517/cdr.2023.152

**Published:** 2024-02-28

**Authors:** Shahil Alam, Pankaj Kumar Giri

**Affiliations:** Faculty of Life Sciences and Biotechnology, South Asian University, New Delhi 110068, India.

**Keywords:** Ovarian cancer, drug resistance, nuclear receptor, non-coding RNA, ovarian cancer stem cells

## Abstract

Ovarian cancer (OC) ranks as the fifth leading factor for female mortality globally, with a substantial burden of new cases and mortality recorded annually. Survival rates vary significantly based on the stage of diagnosis, with advanced stages posing significant challenges to treatment. OC is primarily categorized as epithelial, constituting approximately 90% of cases, and correct staging is essential for tailored treatment. The debulking followed by chemotherapy is the prevailing treatment, involving platinum-based drugs in combination with taxanes. However, the efficacy of chemotherapy is hindered by the development of chemoresistance, both acquired during treatment (acquired chemoresistance) and intrinsic to the patient (intrinsic chemoresistance). The emergence of chemoresistance leads to increased mortality rates, with many advanced patients experiencing disease relapse shortly after initial treatment. This review delves into the multifactorial nature of chemoresistance in OC, addressing mechanisms involving transport systems, apoptosis, DNA repair, and ovarian cancer stem cells (OCSCs). While previous research has identified genes associated with these mechanisms, the regulatory roles of non-coding RNA (ncRNA) and nuclear receptors in modulating gene expression to confer chemoresistance have remained poorly understood and underexplored. This comprehensive review aims to shed light on the genes linked to different chemoresistance mechanisms in OC and their intricate regulation by ncRNA and nuclear receptors. Specifically, we examine how these molecular players influence the chemoresistance mechanism. By exploring the interplay between these factors and gene expression regulation, this review seeks to provide a comprehensive mechanism driving chemoresistance in OC.

## INTRODUCTION

Ovarian cancer (OC), ranking fifth in global women’s mortality, recorded 313,959 incidences and 207,252 deaths^[1]^. Survival rates at 5 years for stages I-IV are 92.4%, 72.9%, 72.9%, and 31.5%, respectively^[2]^. Early-stage diagnosis is challenging, with only 20% identified at stage I, while 70% are discovered at higher stages. The International Federation of Gynaecology and Obstetrics (FIGO) classifies OC based on spreading, with 5-year survival rates of 93%, 75%, and 31% for localized, regional, and distant cases, respectively^[3]^. Epithelial OC (EOC), causing 90% of cases, is a major OC death contributor. The correct staging of OC determines the specific treatment because it provides information about how many cancerous cells are present in body and their location, and cytoreductive surgery followed by chemotherapy is employed in most cases as a treatment strategy. Chemotherapy includes platinum-based drugs such as cisplatin or carboplatin in combination with taxane, generally paclitaxel, and they often develop resistance during chemotherapy or after a few months of the last chemotherapy. The cisplatin reacts N7 of deoxy-guanosine and deoxy-adenine (with low affinity) to form intrastrand, and interstrand crosslink leading to DNA replication blocks, and transcription to induce cell death, while paclitaxel induces cell death via preventing tubulin depolymerization through microtubules stabilization by interacting with β-subunit of tubulin leading to cell cycle arrest during anaphase which required separation of sister chromatids^[4,5]^. Each drug treatment course is termed a cycle, and six cycles of chemotherapy are provided, with each cycle being 3 weeks in length. The neoadjuvant chemotherapy, in which chemotherapy is given both before and after surgery, provides better responses and high 5-year relative survival rates^[6]^. The primary challenge in OC is early-stage detection, hindered by the absence of biomarkers and the asymptomatic nature in the initial stages. Another critical issue is chemotherapy-related mortality, wherein patients develop acquired chemoresistance or exhibit intrinsic chemoresistance. Initially, 70% respond to platinum and taxane-based therapies, but resistance develops via several mechanisms such as alteration of drug efflux/influx, increased antioxidant to neutralize reactive oxygen species (ROS) generated due to platinum-based drugs, decreased apoptosis, and hyperactive DNA repair contributing to increased mortality and relapse within 2 years for many patients^[7-9]^. Understanding chemoresistance involves complex mechanisms with poorly understood regulation of gene expression involved in chemoresistance mechanisms. Factors like ncRNA and nuclear receptors influencing chemoresistance lack comprehensive exploration, making them vital areas for further study. This review delves into chemoresistance related to transport systems, apoptosis, DNA repair, and OCSCs. It scrutinizes the modulation of key cellular processes that foster chemoresistance, encompassing efficient DNA repair, efflux transporter upregulation, OCSCs proliferation, apoptosis inhibition, and influx transporter downregulation. The discussion extends to the role of ncRNA and nuclear receptors for regulating genes associated with various chemoresistance mechanisms in OC.

## THE ROLE OF APOPTOSIS IN CHEMORESISTANCE IN OC

This segment of the review explores the intricate interconnection between drug resistance and apoptosis within the context of OC. In our thorough examination, we have delved into the pivotal modulators affected under both sensitive and resistant conditions. Under sensitive conditions, our review highlights key modulators, such as apoptotic protein mechanisms, that play essential roles in maintaining cellular homeostasis and preventing tumorigenesis. Conversely, in resistance conditions, these modulators undergo alterations, compromising their responsiveness to therapeutic interventions. Our comprehensive review has methodically summarized the modifications in these key modulators, elucidating their significance in mediating drug resistance in OC.

**Figure 1:**
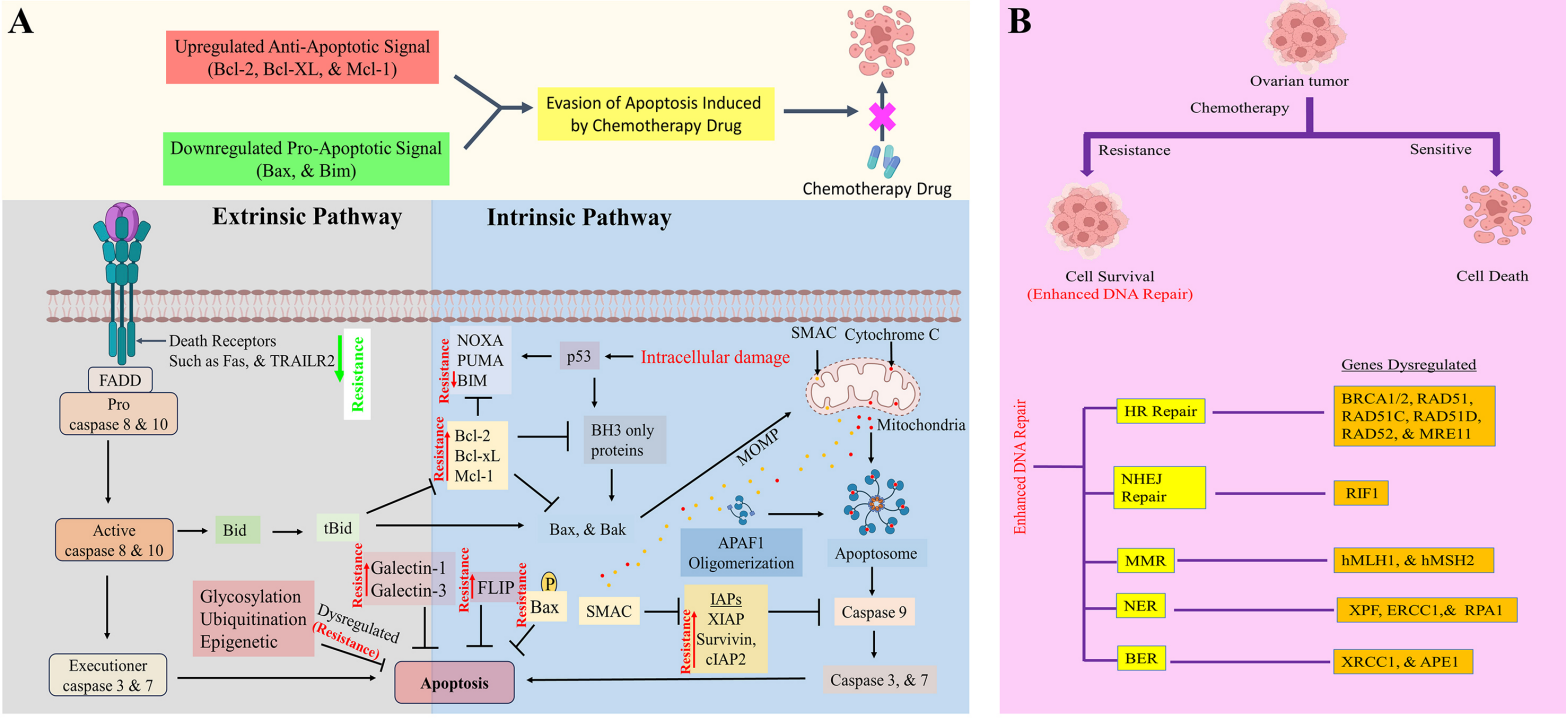
(A) Apoptosis modulation to enhance chemoresistance in OC. The increased expression of Bcl-2, MCL-1, Bim, Bcl-XL, IAPs (Survivin, XIAP, &cIAP2), Bax phosphorylation and apoptosis regulation by ubiquitination, galectin, glycosylation, and epigenetic regulation inhibit apoptosis to confer chemoresistance in OC; (B) Increased DNA repair activity to enhance chemoresistance in OC. The aberrant expression of DNA repair genes, i.e., BRCA1/2, RAD51, and its paralogs RAD5IC and RAD5ID, RAD52, MRE11, RIFI, hMLH1, hMSIH2, ERCC1, XPF, RPA1, APE1, and XRCC1, promotes drug resistance in OC. Created with BioRender.com. OC: Ovarian cancer; Bcl-2: B cell lymphoma gene 2; MCL-1: myeloid cell leukemia sequence 1; IAPs: inhibitors of apoptosis proteins; X-linked inhibitor of apoptosis protein; BRCA 1/2: breast cancer gene 1/2; MRE11: meiotic recombination 11; hMLH1: human MutL homolog 1; hMSH2: human MutS homolog 2; ERCC1: excision repair cross-complementation group 1; XPF: xeroderma pigmentosum complementation group F; XRCC1: X-ray repair cross-complementing 1.

For convenience and improved understanding, we have compiled an extensive table [Table 1] detailing the observed alterations in key modulators under both sensitive and resistant conditions. Additionally, we have created a comprehensive figure [Figure 1A] that visually represents the intricate pathways associated with drug resistance in OC. This schematic diagram is designed to enhance your comprehension of the intricate interplay between apoptosis and drug resistance mechanisms.

**Table 1:** The role of apoptosis in chemoresistance in OC

		**Effector molecule**	**Differential expression profile**	**Model system**	**Chemotherapy agents**	**Associated-mechanism**	**Ref.**
**Intrinsic pathway apoptosis**	**Anti-apoptotic**	Bcl-2	Up	SKOV3 spheroid	Cisplatin	-	[10]
				SKOV3	Paclitaxel	PKR-Bcl2	[11]
		Bcl-XL	Up	SKOV3	Cisplatin	-	[12]
		MCL-1	Up	OVCAR3, A2780	Carboplatin	MGMT-DUB3-MCL1	[13]
	**Pro-apoptotic**	Bax	-	MDA‐MB‐468	ABT‐737	Akt-Bax	[14]
		Bim	Down	ES2, TOV21G, SKOV3, OVTOKO	ABT-263	ZEB1-BIM	[15]
**Extrinsic pathway apoptosis**		Membrane TRAIL-R2	Down	OAW42, SKOV3, A2780	TRAIL	CHKA-TRAIL-R2	[16]
		FLIP	Up	OV2008, C13	Cisplatin	-	[17]
**IAPs mediated apoptosis**		XIAP	Up	SKOV3	Docetaxel	-	[18]
				A2780, BALB/c nude mice	Cisplatin	-	[19]
		Survivin	Up	IGROV-1, OAW42	Taxol	-	[20]
		cIAP2	Up	SKOV3, OVCAR3	Cisplatin	Il-6-cIAP2	[21]
**Ubiquitination mediated apoptosis**		EDD/UBR5	Up	A2780ip2, OVCAR5, ES-2	Cisplatin	EDD-Mcl-1	[22]
				A2780 and Tyknu	Cisplatin	EDD/Dyrk2-MOAP-1	[23]
		ITCH	-	OV2008, A2780s	Cisplatin	FLIP-p53-Itch	[24]
		HOIP	Up	A2780	Cisplatin	JNK pathway	[25]
		CRL4	Up	A2780	Cisplatin	CRL4-STAT3-BIRC3	[26]
**Glycosylation mediated apoptosis**		ST6Gal1	Up	A2780	Cisplatin	-	[27]
		HSPG(Syndecan-2)	Up	SKOV-3, OVCAR-3	Cisplatin	DcR3-HSPG	[28]
		N-Glycosylation	-	OVCAR-3	Tunicamycin	ER-stress	[29]
**Galectin mediated apoptosis**		Galectin-1	Up	A2780/CP	Cisplatin	H-Ras/Raf-1/ERK pathway, p21, Bcl-2	[30]
		Galectin-3	Up	OVCAR-3	Cisplatin	Mitochondrial dysfunction	[31]
**Epigenetic mediated apoptosis**		hMOF	Up	OVCAR3/DDP	Cisplatin	hMOF-MDM2	[32]
		DNA methyltransferase inhibitors decitabine	-	platinum-resistant OC patients	Carboplatin	-	[33]
		HDAC inhibitors entinostat, avelumab	-	Recurrent OC patient	Platinum	-	Clinical trail (NCT02915523)

Bcl-2: B cell lymphoma gene 2; PKR: protein kinase R; MGMT: O6-methylguanine-DNA methyltransferase; DUB3: deubiquitinating enzyme 3; ZEB1: zinc finger E-box-binding homeobox 1; CHKA: choline kinase-α; FLIP: fas-associated death domain-like interleukin-1β-converting enzyme (FLICE)-like inhibitory protein; IAPs: inhibitors of apoptosis proteins; UBR5: ubiquitin protein ligase E3 component N-recognin 5; DYRK2: dual-specificity tyrosine phosphorylation-regulated kinase 2; MOAP-1: modulator of apoptosis 1; ITCH: itchy E3 ubiquitin protein ligase; HOIP: HOIL-1L interacting protein; CRL4: cullin 4-RING ubiquitin ligase; ST6Gal1: beta-galactoside alpha-2,6-sialyltransferase 1; hMOF: human males absent on the first; MDM2: murine double minute 2.

## THE ROLE OF DNA REPAIR IN CHEMORESISTANCE IN OC

The role of DNA repair mechanisms in drug resistance within OC is a critical aspect of understanding the challenges and complexities associated with treatment. DNA repair processes have a significant role in the response of cancerous cells to various therapeutic agents^[34]^. Understanding the intricate relationship between DNA repair mechanisms and drug resistance in OC is crucial for formulating targeted therapies that can overcome or exploit these mechanisms, ultimately improving treatment outcomes for patients with this challenging disease. To address this complexity, we have chosen a strategic approach to streamline and simplify the information for our audience. We condensed the details concerning the role of drug resistance on DNA repair pathways into an extensive table [Table 2]. This tabular format serves as a comprehensive reference, presenting the main insights and discoveries in an organized manner. It offers a concise yet informative overview of the subtle interactions within the DNA repair pathways influenced by drug resistance in OC. Additionally, alongside the table, we have created an illustrative figure [Figure 1B], that visually illustrates the impact of drug resistance on DNA repair pathways in OC. This visual representation aims to enhance comprehension of the intricate relationships between drug resistance mechanisms and the complex processes of DNA repair.

**Table 2:** Overview of the DNA repair mechanisms contributing to chemoresistance in OC

**DNA repair pathway**	**Gene**	**Differential expression profile**	**Model system**	**Chemotherapy agent**	**Associated-mechanism**	**Ref.**
HR	*BRCA1*	-	Recurrent OC	Platinum	Reversion mutation, increased loss of methylation	[35]
	*BRCA1/2*		Recurrent OC	Platinum, PARPi	Somatic mutation	[36]
	*RAD51*	Up	CP70 and SKOV3	PARPi	LCK-RAD51/BRCA1/2	[37]
	*RAD51C*, *RAD51D*	-	OC patient	PARPi	Secondary mutation	[38]
	*RAD52*	Up	A2780 cisR	Cisplatin	PAF1/PD2-RAD52	[39]
	*MRE11*	-	COV362	PARPi	DYNLL1-MRE11	[40]
NHEJ	*RIF1*	Up	EOC patients	Cisplatin	-	[41]
NER	*ERCC1-XPF*	Up	A2780	Cisplatin	-	[42]
	*ERCC1*	Up	OC patient	Platinum	-	[43]
	*RPA1*		OVCAR8	Camptothecin	DOCK7-RPA1	[44]
BER	*XRCC1*	Up	OVCAR-3, OVCAR-4	Cisplatin	-	[45]
		-	SKOV3/DDP	Cisplatin	HSP90-XRCC1	[46]
		-	OC patient	Cisplatin	XRCC1 194 Trp/Trp, XRCC1 399Arg/Arg polymorphism	[47]
	*APE1*	Up	OC patient	Platinum	-	[48]
MMR	*hMLH1*	-	A2780/cp70	Cisplatin	Hypermethylation	[49]
	*hMSH2*	-	A2780	Cisplatin	Hypermethylation	[50]

OC: Ovarian cancer; HR: homologous recombination; BRCA 1/2: breast cancer gene 1/2; PARPi : poly (ADP-ribose) polymerase inhibitor; LCK: lymphocyte-specific protein tyrosine kinase; PAF1: RNA polymerase II-associated factor 1; DYNLL1: dynein light chain LC8-type 1; MRE11: meiotic recombination 11; NHEJ: non-homologous end joining; RIF1: replication timing regulatory factor 1; NER: nucleotide excision repair; ERCC1: excision repair cross-complementation group 1; XPF: xeroderma pigmentosum complementation group F; DOCK7: dedicator of cytokinesis 7; BER: base excision repair; XRCC1: X-ray repair cross-complementing 1; MMR: mismatch repair; hMLH1: human MutL homolog 1; hMSH2: human MutS homolog 2.

## THE ROLE OF TRANSPORT SYSTEM IN CHEMORESISTANCE IN OC

**Figure 2:**
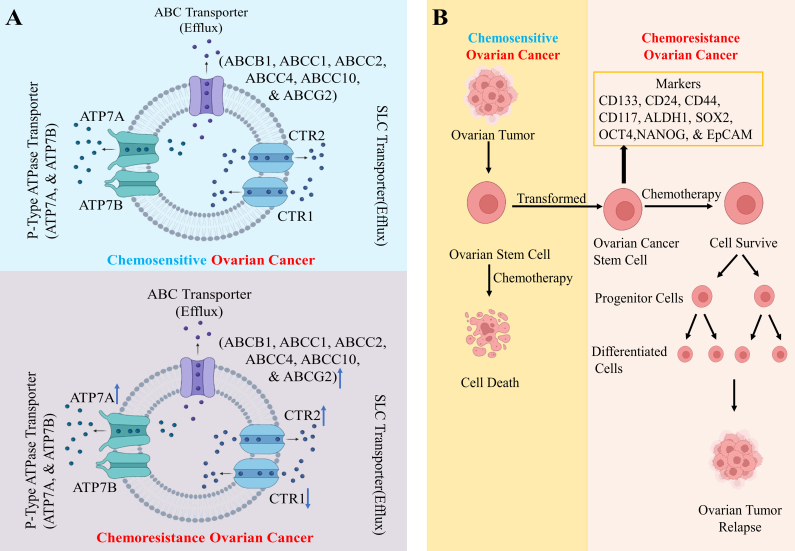
(A) Schematic diagram of drug transport in chemoresistance in OC. ABC transporters such as ABCB1, ABCC1, ABCC2, ABCC4, ABCC10, and ABCG2, P-type ATPase ATP7A, and SLC transporter CTR2 responsible for drug efflux are upregulated, while SLC transporter CTR1 that functions for drug influx is downregulated, and P-type ATPase transporter ATP7B does not directly facilitate drug efflux, but its genetic polymorphism can influence drug efflux; (B) OCSCs confer chemoresistance in OC. OSCs are transformed into OCSCs due to genetic alteration, which then proliferate into progenitor cells and subsequently differentiate into cells that contribute to the relapse of ovarian tumors after some time instead of undergoing cell death upon chemotherapy. Created with BioRender.com. OC: Ovarian cancer; ABCB1: ATP-binding cassette subfamily B member 1; ABCC: ATP-binding cassette subfamily C; ABCG2: ATP-binding cassette subfamily G member 2; SLC: solute carrier; CTR1/2: copper transporter 1/2; OCSCs: ovarian cancer stem cells.

The optimal effectiveness of a drug within cells depends on its optimal cytoplasmic concentration. Membrane transporters, particularly ABC transporter, P-type ATPase transporter, and solute carrier (SLC) transporter, are key factors influencing the bioavailability of drugs in ovarian cancer [Figure 2A and Table 3].

**Table 3:** The role of transport system in chemoresistance in OC

		**Differential expression profile**	**Model system**	**Chemotherapy agent**	**Associated-mechanism**	**Ref.**
**ABC superfamily transporters**	ABCB1	Up	A2780, SKOV3	Cisplatin	SORL1-EEA1-ABCB1	[51]
					Gli2-ABCB1	[52]
	ABCC1	Up	OC patient	Cisplatin, paclitaxel	-	[53]
	ABCC2	Up	A2780, SKOV3	Cisplatin	HnRNPA2B1-ABCC2	[54]
	ABCC4	Up	27/87	Topotecan	MYCN-ABCC4	[55]
	ABCC10	Up	SKOV3	Paclitaxel, docetaxel, vincristine, vinorelbine	-	[56]
	ABCG2	Up	OVCAR-3 S, CAOV-3 S	Adriamycin	HIF-2α-ABCG2	[57]
**P-type ATPase superfamily transporter**	ATP7A	Up	A2780-CP20	Cisplatin	-	[58]
			OC patient	Cisplatin	Polymorphism	[59]
	ATP7B	Up	A2780-CP20	Cisplatin	-	[60]
			IGROV-CP20	Cisplatin	TFEB-ATP7B	[61]
**SLC superfamily transporters**	CTR1	Down	A2780-CP	Cisplatin	-	[62]
			A2780	Cisplatin	Core fucosylation	[63]
	CTR2	Up	OV-2008	Cisplatin	-	[64]

OC: Ovarian cancer; ABC: ATP-binding cassette; SORL1: sortilin related receptor 1; EEA1: early endosome antigen 1; HnRNPA2B1: heterogeneous nuclear ribonucleoproteins A2/B1; ABCG2: ATP-binding cassette subfamily G member 2; HIF-2α: hypoxia inducible factor 2 alpha; SLC: solute carrier; CTR1/2: copper transporter 1/2.

### ABC superfamily transporters

ABC transporters, with seven families, use ATP to facilitate substrate efflux/influx. Comprising 49 members and 21 pseudo-members, they mainly efflux substrates. Structurally, ABC transporters have single polypeptides with two nucleotide binding domains (NBDs) and two transmembrane domains (TMDs)^[65]^. The SNAIL, ZEBs, and SLUG promote MDR, while VDR, ER, PXR, KLF, Gli, and Sp are also known to modulate ABC transporters^[66]^. The altered ABCB1 structure or drug binding, inhibition of its expression or knockout of the ABCB1 gene are the most potential strategies to overcome ABCB1-mediated drug resistance^[67]^. Similarly, Basic helix-loop-helix family member e40 (BHLHE40) is inversely related to ABCB1, suggesting that the upstream target of ABCB1 can be used to overcome ABCB1-mediated chemoresistance^[68]^. Recent research has explored the broad substrate specifity, and conversion of efflux to influx pump via engineering of ABC transporter, and the importance of membrane transporters is also highlighted in the development of precision medicine^[69,70]^.

ABCB1 elevation resists cisplatin/paclitaxel and knockdown restore sensitivity^[71-74]^. Paclitaxel, a direct substrate of ABCB1, regains sensitivity upon ABCB1 mutation, while 14 residues replacement in helices 6 and 12 reverses ABCB1’s efflux to influx of taxol derivative Flutax-1^[75,76]^. Sortilin-related receptor 1 (SORL1) silencing inhibits the endosomal antigen 1 pathway, delaying ABCB1 stabilization, sensitizing cis-diamminedichloroplatinum(II) (CDDP)-resistant ovarian cells^[77-79]^.

ABCB1 is regulated by the Hedgehog pathway, with Gli2 directly targeting and positively modulating its expression^[80]^. ABCB1 Single Nucleotide Polymorphism (SNP), 3435C>T, enhances docetaxel efflux in OC^[81]^. ABCC1, ABCC2, and ABCC4 are associated with chemoresistance. ABCC1 has significantly elevated levels in primary drug (cisplatin and paclitaxel) resistant EOC tissues^[82,83]^. Heterogeneous nuclear ribonucleoprotein A2/B1 (HnRNPA2B1) affects ABCC2 translation, blocked by interferon-stimulated gene 15 (*ISG15*), downregulated in cisplatin-resistant ovarian cancer^[84]^. ABCC4 confers resistance to topotecan and irinotecan in high myc expression OC^[85]^.

ABCC10 overexpressed in established SKOV3 cell line resistance to paclitaxel, docetaxel, vincristine, and vinorelbine enhanced epithelial to mesenchymal transition (EMT) in OC, restored by cepharanthine, ABCC10 inhibitor^[86]^. ABCG2, elevated in topotecan-resistant A2780 cells, regains sensitivity with ABCG2 antagonists. Hypoxia inducible factor 2 alpha (HIF-2α) upregulation promotes OCSC stemness and ABCG2-mediated Adriamycin resistance^[87]^.

### P-type ATPase superfamily transporter

P-type ATPase superfamily transporter, with five subfamilies (P1-P5), transports ions across membranes using ATP hydrolysis energy. P-type ATPases have cytosolic domains (A, P, N) and transmembrane domains (M1-M6, with P1 having M7-M10)^[88]^. ATP7B knockdown enhances cisplatin sensitivity. ATP7A silencing lacks impact on resistance, but ATP7A polymorphism is linked to cisplatin resistance in ovarian cancer^[89]^. The transcription factor EB (TREB) binds the promoter's first intron region at coordinated lysosomal expression and regulation (CLEAR) sites of ATP7B, modulating its expression when exposed to platinum drugs in ovarian cancer^[90]^.

### SLC superfamily transporters

SLC transporters, classified into 65 families based on sequence similarity, generally uptake substrates but can be bidirectional or efflux. They exist as homodimers or heterodimers^[91]^. Cisplatin-sensitive A2780 shows higher copper transporter 1 (CTR1), while cisplatin-resistant has reduced uptake due to CTR1 downregulation upon exposure^[92,93]^. The CTR1 core fucosylation is higher in A2780 resistant, suppressing CTR1-CDDP interaction and affecting cisplatin uptake^[94]^. Copper transporter 2 (CTR2) downregulation increases cisplatin sensitivity, linked to platinum efflux^[95]^.

## THE ROLE OF OCSC IN CHEMORESISTANCE IN OC

New research indicates that contrary to previous beliefs about a constant follicle in the ovary at birth, there are ovarian stem cells (OSCs) present. These include a dormant group of very small embryonic-like stem cells (VSELs) and a larger subset of dividing OSCs. OSCs are undifferentiated cells inherently capable of self-renewal, proliferation, multipotency, and differentiation. VSELs, which express embryonic markers such as octamer-binding transcription factor 4 (OCT-4), are located in ovary surface epithelium, and can divide asymmetrically to self-renew to form OSC^[96]^. OSCs are maintained by a niche microenvironment composed of ECM, immune cells, stromal cells, mesenchymal cells, and vascular network^[97]^. A recent study identified the tubal-peritoneal junction & hilum region as stem cell niche within the ovary^[98]^. Ovarian tumors exhibit heterogeneity with distinct cell types expressing stem cell markers cluster of differentiation 133 (CD133), leucine-rich repeat-containing G-protein coupled receptor 5 (LGR5), aldehyde dehydrogenase 1 (ALDH1), and cytokeratin 6B (CK6B). These cells, known as OCSCs, arise from genetic instability in OSCs, contributing to chemoresistance, cancer initiation, and treatment failure^[98]^. OCSC, characterized by various markers, may display diverse phenotypes, offering selective advantages^[99]^. OCSCs inherently resist chemotherapy. Targeting OCSC and pathways offers strategies against OC chemoresistance^[99]^ [Figure 2B and Table 4].

**Table 4:** The role of OCSC in chemoresistance in OC

**OCSC marker**	**Differential expression profile**	**Chemotherapy agent**	**Associated-mechanism**	**Ref.**
ALDHA1A1	Up	Carboplatin	RAD6-H2B-SOX2	[100]
SOX2	Up	Carboplatin	RAD6-H2B-SOX2	[100]
		Cisplatin, paclitaxel	-	[101]
		-	Hypoxia-notch-SOX2	[102]
		-	SOX2- to β-catenin	[103]
CD133	Up	-	NF-κB, MAPK pathway	[104]
NANOG	Up	Cisplatin, paclitaxel	-	[105]
OCT4	Up	Cisplatin, paclitaxel	-	[105]
		-	AKT pathway	[106]
CD44	Up	Cisplatin, paclitaxel	AKT pathway	[106]
EpCAM	Up	Doxorubicin	-	[107]
ALDH1	Up	Platinum	-	[108]
CD24	Up	Cisplatin	EMT	[109]
CD133	Up	Cisplatin, paclitaxel	-	[110]
CD177	Up	Cisplatin, paclitaxel	CD177-ABCG2	[111]
HLF	Up	Carboplatin	HLF-YAP1	[112]

OCSC: Ovarian cancer stem cell; SOX2: SRY-box transcription factor 2; MAPK: mitogen-activated protein kinase; OCT4: octamer-binding transcription factor 4; AKT: Ak strain transforming; ALDH1: aldehyde dehydrogenase 1; EMT: epithelial to mesenchymal transition; ABCG2: ATP-binding cassette subfamily G member 2; HLF: hepatic leukemia factor.

### OCSC markers

SRY-box transcription factor 2 (SOX2) overexpression induces cisplatin, carboplatin, and paclitaxel resistance in OC, and higher Nanog Homeobox (NANOG) and OCT-4 also found to confer cisplatin and paclitaxel resistance in OCSCs^[113]^. Overexpression of c-kit, NANOG, ABCG2, ABCG5, and MDR1 is implicated in OCSC maintenance^[114]^. CD133 promotes OCSC stemness, increasing cisplatin and paclitaxel resistance in OC, and Epithelial cell adhesion molecule (EpCAM) overexpression is stimulated with doxorubicin promoting stemness in SKOV3 and OVCAR5^[115,116]^. The enrichment of OCSC markers ALDH1 is found in OC patients after platinum-based chemotherapy treatment^[117]^. RAD6 upregulation in chemoresistant OC mediates histone 2B ubiquitination, regulating stem cell genes (ALDH1A1 and SOX2). Silencing RAD6 reduces DNA repair signaling and OCSC markers, sensitizing OC to carboplatin^[117]^. CD117 expression correlates with chemotherapy response, with CD44+CD117+ cells exhibiting cisplatin and paclitaxel resistance compared to CD44-CD117- cells. CD177+ cells overexpress ABCG2, conferring cisplatin and paclitaxel resistance, while CD24 expression induces EMT and cisplatin resistance in OC OC^[117,118]^. CD44 and ALDH1A1+ OCSCs confer platinum and taxane resistance, with ALDH1 silencing sensitizing cells to chemotherapy^[119-122]^. The platinum treatment induces OCSC proliferation, blocked by a combination of platinum and oxidative phosphorylation (OXPHOS) inhibitors^[123]^.

### OCSCs signaling pathways

The activated Wnt/β-catenin pathway activation promotes the OCSC and STAT3 also promotes the stemness through the Wnt/β-catenin pathway^[124]^. The SOX2 elevated in OCSC binds to β-catenin to sustain stem cell-like features of OCSCs^[124]^. Calcitriol decreases OCSCs through downregulation of Wnt/β-catenin^[124]^. NF-κB and MAPK signaling pathways promote CD133+ OCSCs^[124]^. DAPT inhibits Notch signaling, reducing OCSC self-renewal and Notch3 silencing enhances platinum sensitivity^[124]^. Galectin-3 activates Notch1, maintaining OCSCs^[124]^. The H/ACA box 72 (SNORA72) promotes OCSC activation via Notch1-Cellular-Myc (c-Myc) axes^[125]^. Notch3 upregulation in recurrent tumors suggests a role in tumor relapse^[126]^. The PI3K/AKT pathway enhances OCSC markers, conferring cisplatin and paclitaxel resistance in OC^[127]^. Akt inhibition (NV-128) induces apoptosis in CD44+/Myeloid Differentiation Primary Response 88 (MyD88)+ OCSCs via Reactive ROS-dependent ERK activation. 2-(4-morpholinyl)-8-phenyl-chromone (LY294002) suppresses Oct 4, ABCG2, and P-gp in SKOV3 OCSCs, potentially reducing chemoresistance^[127]^. Hepatic leukemia factor (HLF) upregulation in OCSCs maintains OCSCs and confers carboplatin resistance. Mechanistically, HLF activates YAP1 expression, modulating the Hippo signaling pathway. Silencing HLF or using the YAP1 inhibitor verteporfin attenuates carboplatin resistance in OC^[128]^.

### The interplay between non-coding RNA and OCSCs

The non-coding RNAs are RNA molecules that are transcribed but not translated, and categorized based on length and shape, such as circular RNA (circRNA) with a circular structure, long non-coding RNA (lncRNA) exceeding 200 nucleotides, and microRNA (miRNA) with an average length of 22 nucleotides^[129]^. The lncRNA, miRNA, and circRNA are found to regulate the OCSCs, leading to tumor relapse and chemotherapy resistance^[130]^. The lncRNA SNORD89 is highly upregulated in OCSCs and promotes its stemness by upregulating the Notch1-c-Myc pathway^[131]^. Similarly, the silencing of another lncRNA MALAT1 decreases the OC cell stemness and increases the cisplatin sensitivity through interaction with yes-associated protein (YAP), blocking its movement from the nucleus to the cytoplasm and bolstering the stability of the YAP^[132]^. lncRNA HOTAIR increased in OCSCs to confer cisplatin resistance and its depletion resulted in reduced resistance to cisplatin in OCSCs. Mechanistic study shows HOTAIR promotes the T-box transcription factor 3 (TBX3), and maintains stemness of OC expression by sponging miR-206^[132]^. Using PNA3 to target HOTAIR, thus interrupting its binding with EZH2, along with a DNA methyltransferase (DNMT) inhibitor, leads to a decrease in ALDH+ (OCSCs)^[133]^. The downregulation of lncRNA TUG1 and overexpressed miR-186-5p suppressed OCSCs. A mechanistic study shows that TUG1 sponges miR-186-5p to release ZEB1 to promote the stemness of OC cells. The downregulation of miR-429 and miR-591 targets ZEB1 to confer cisplatin and paclitaxel resistance, respectively, in OC^[133,134]^. lncRNA LINC01234 adsorb miRNA-27b-5p to promote the silent information regulator 5 (SIRT5) expression to induce OCSCs progression^[135]^. The lncRNA XIST is found to be downregulated in OC to confer paclitaxel resistance, and mechanistic study shows that it increases Lysine N-methyltransferase 2C (KMT2C) via targeting miR-93-5p to regulate CD44+/CD24- OSCs^[136]^. lncRNA-H19 sponges miR-29b-3p to promote STAT3 to promote carboplatin-resistant EOC^[137]^. Several miRNAs are also known to regulate the OCSCs. The downregulation of miR-200c promotes Gab2-enhanced expansion of ALDH+(stem cell maker) cells^[138]^. The overexpression of Yin Yang 1 (YY1) promotes the OCSCs via recruiting HDAC5 to the miR-99a, and enhancing the miR-99a deacetylation and decreased miR-99a^[139]^. The miR-26b is downregulated in CD117+CD44+ OCSCs to promote its stemness and mechanistic study shows its functional target is PTEN^[140]^. miR-181a is found to promote stemness and cisplatin resistance in HGSOC via the Wnt/β-catenin pathway^[141]^. miR-600 binds to the 3’-untranslated region of Krueppel-like factor 9 (KLF9) to supress its expression to promotes OCSCs^[141]^.Similarly, miR-181a-2-3p suppresses the stemness of CD44-positive OCSCs through its interaction with EGR1^[142]^. Downregulating miR-21 significantly decreased CD133+ population and cancer stem/progenitor cells (CSPC) sphere formation, while miR-21 overexpression increased CD133+ cells and CSPC spheres^[143]^. circRNA, like circ_0000745, modulated by insulin-like growth factor 2 mRNA-binding protein 2 (IGF2BP2) boosts SKOV3 stemness by sponging miR-3187-3p to enhance ERBB4, thus phosphorylating the PI3K/AKT signalling^[144]^. The circRNA microarray analysis revealed 159 upregulated and 55 downregulated circRNAs in OCSCs, suggesting circular RNA is a crucial player in driving OCSC stemness^[145]^.

## THE ROLE OF NUCLEAR RECEPTORS IN CHEMORESISTANCE IN OC

**Figure 3:**
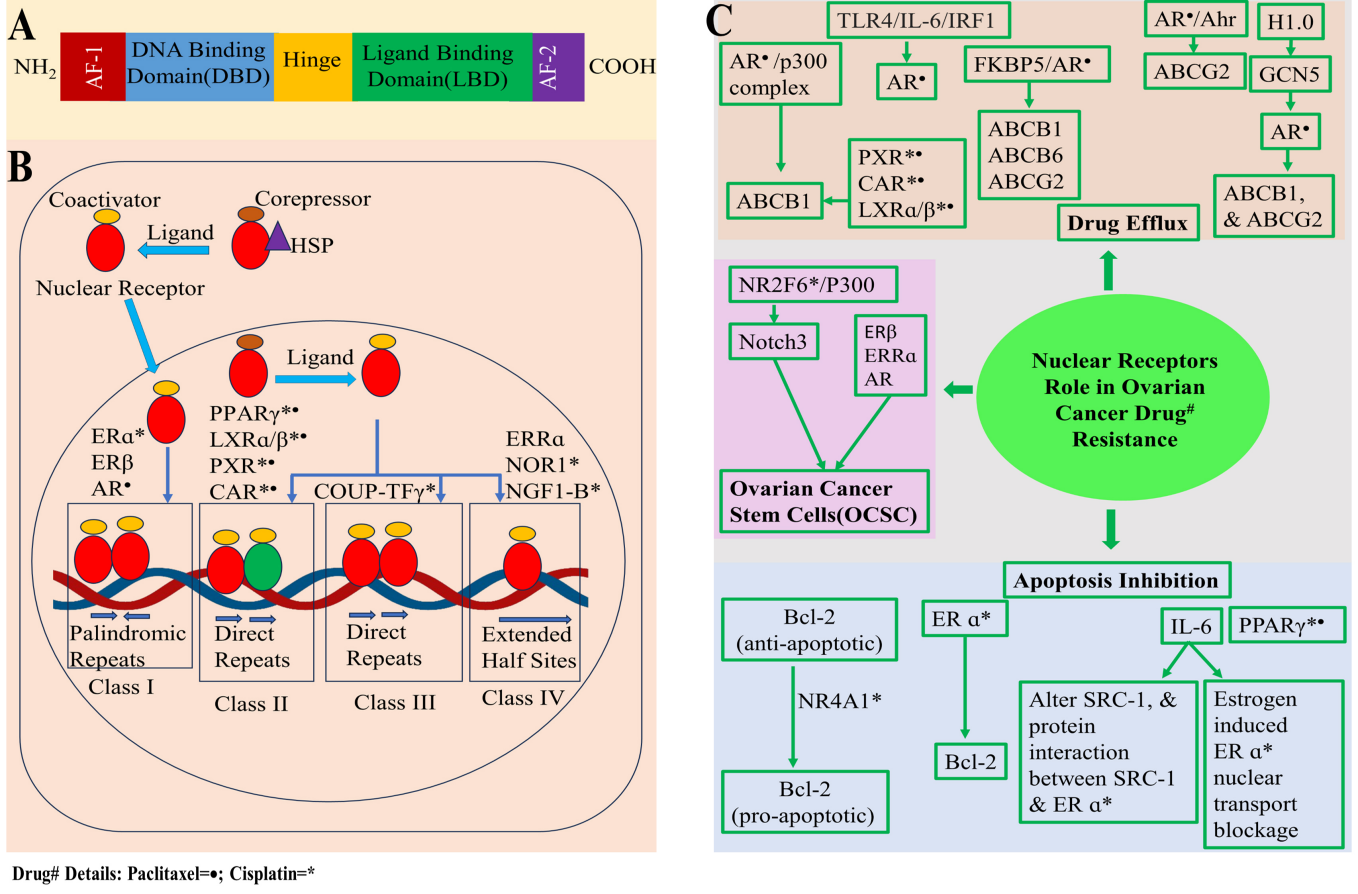
(A) Nuclear receptors domain architecture; (B) Diagram illustrating the categorization of nuclear receptors associated with chemoresistance in OC, with a focus on their interaction with ligands; (C) Demonstrating various mechanisms associated with the involvement of nuclear receptors in OC drug^#^ resistance, primarily encompassing the inhibition of apoptosis, drug efflux, and the presence of OCSCs. Created with BioRender.com. OC: Ovarian cancer; OCSCs: ovarian cancer stem cells.

Nuclear receptors, activated by lipid-soluble signals like steroid hormones, regulate gene expression via hormone response elements (HRE), impacting proliferation, apoptosis, and metabolism^[146]^. There are 48 nuclear receptors in humans, and when their normal functioning is disrupted, it is frequently associated with various diseases. Nuclear receptors are classified into seven families, i.e., NR0-6, based on sequence homology^[147]^. Nuclear receptors are structured into four segments: the unstructured N-terminal domain (NTD) housing activation function 1 (AF-1), DNA binding domain (DBD), Hinge region, and ligand binding domain (LBD). The LBD binds to ligands and engages with co-regulator proteins via activation function 2 (AF-2) [Figure 3A]^[147]^. The nuclear receptor co-regulators are divided into two categories, i.e., coactivators and corepressors, which directly interact with AF-1 and AF-2 regions of nuclear receptors. Coactivators bind via LXXLL motifs, and corepressors via CoRNR box motifs^[147]^. Nuclear receptors are also classified into classes I, II, III, and IV based on ligand binding and DNA binding [Figure 3B]. Class I nuclear receptors are sequestered in cytoplasm with chaperon proteins, but upon ligand (cholesterol-derived steroidal hormones) activation, they enter inside the nucleus to bind DNA response elements (RE) composed of two inverted repeats as homodimers^[147]^. Class II nuclear receptors are present in nucleus with corepressor, but upon ligand activation, corepressor is swapped with coactivators and binds to DNA RE consisting of direct repeat sequence as heterodimers^[147]^. Class III nuclear receptors are similar in working mechanism to class II, Table 5 except they bind to DNA RE comprising direct repeat sequence as homodimers^[147]^. Class IV are also similar to the working mechanism of class II, except they bind to extended half-sites within DNA RE as monomers^[147]^. Chemotherapy is among the top three commonly used treatments for OC. Unfortunately, its effectiveness is restricted by OC cells that have become resistant to the drugs^[148]^. The importance of various nuclear receptor families in controlling drug metabolism and distribution is gaining recognition, and therapies aimed at these receptors offer new possibilities to mitigate or potentially prevent drug resistance^[149]^. In this context, we will explore the latest findings concerning the roles and control of different nuclear receptors in the emergence of drug resistance in OC. We will also shed light on how nuclear receptors are linked to drug resistance during chemotherapy and nuclear receptors associated with chemoresistance in OC [Table 5 and Figure 3C].

**Table 5:** Overview of different nuclear receptors implicated in drug resistance in OC, covering specific drug, differential expression patterns, and their associated mechanisms

**Family of NRs**	**Class** **of NRs**	**Gene name**	**Common name**	**Differential expression profile**	**Chemotherapy agent**	**Target-gene**	**Associated-mechanism**	**Ref.**
1C	II	*PPARG*	PPAR γ	Down	Cisplatin; paclitaxel	-	Apoptosis inhibition	[150]
1H	II	*NR1H3/NR1H2*	LXR α/LXR β	Up	Cisplatin; paclitaxel	*MDR1*	Drug efflux	[151]
1I	II	*NR1I2*	PXR	Up	Cisplatin; paclitaxel	*MDR1*	Drug efflux	[152]
1I	II	*NR1I3*	CAR	Up	Cisplatin; paclitaxel	*MDR1*	Drug efflux	[153]
2F	III	*NR2F6*	COUP-TF γ	Up	Cisplatin	*Notch3*	OCSCs	[154]
3A	I	*ESR1*	ER α	Up	Cisplatin	*Bcl-2*	Apoptosis inhibition	[155]
3A	I	*ESR2*	ER β	Up	-	*SOX2*, *OCT4*	OCSCs	[156]
3B	IV	*ESRRA*	ERR α	Up	-	-	OCSCs	[157]
3C	I	*AR*	AR	Up	Paclitaxel	*ABCB1*, *ABCB6*, *ABCG2*; *AR/Ahr-ABCG2*; *TLR/IL-6/IRF1*; *NANOG*	Drug efflux; OCSCs	[158-164]
4A	IV	*NR4A1*	NGF1-B	Down	Cisplatin	*Bcl-2*	Apoptosis inhibition	[165]
4A	IV	*NR4A3*	NOR1	Up	Cisplatin	-	-	[166]

OC: Ovarian cancer; NR: nuclear receptor; PPARG: peroxisome proliferator-activated receptor gamma; PPAR: peroxisome proliferator activated receptor; NR1H3: nuclear receptor subfamily 1 group H member 3; NR1H2: nuclear receptor subfamily 1 group H member 2; LXR: liver X receptor; NR1I2: nuclear receptor subfamily 1 group I member 2; PXR: pregnane X receptor; CAR: constitutive androstane receptor; NR2F6: nuclear receptor subfamily 2 group F member 6; COUP-TF: chicken ovalbumin upstream promoter-transcription factor; Notch3: neurogenic locus notch homolog protein 3; OCSCs: ovarian cancer stem cells; ESR1: estrogen receptor 1; ER: estrogen receptor; SOX2: SRY-box transcription factor 2; AR: androgen receptor; ABCB6: ATP-binding cassette subfamily B member 6; ABCG2: ATP-binding cassette subfamily G member 2; NR4A1: nuclear receptor subfamily 4 group A member 1; NGF1-B: nerve growth factor 1B; NR4A3: nuclear receptor subfamily 4 group A member 3; NOR1: neuron-derived orphan receptor 1.

PPARγ inhibits apoptosis, while LXRα/β upregulates cholesterol and MDR1 for drug efflux to confer cisplatin and paclitaxel resistance in OC^[167,168]^. PXR and CAR activation boosts cisplatin and paclitaxel resistance and downregulation suppress MDR1/ABCB1, enhancing apoptosis^[169-171]^. ERα and androgen receptor (AR) from family 3 contribute to chemoresistance, with ERα inducing cisplatin resistance in OC^[172]^. Tamoxifen resistance is associated with IL-6, impacting ERα, ERβ, and steroid receptor coactivator-1 (SRC-1) expression and inhibition of estrogen-induced ER nuclear translocation^[173,174]^. FK506 binding protein 5 (FKBP5) silencing sensitizes OC to taxol, forming a complex with AR and modulating taxol resistance genes ABCB1, ABCB6, and ABCG2^[175]^. Taxol activates the Akt pathway, triggering p300-mediated increases in AR expression and chromatin remodeling of the ABCB1 gene. This involves AR/H3K9ac and AR/H3K14ac interactions, followed by AR and p300 binding at the androgen-response elements (ARE4) of ABCB1, leading to heightened expression and chemoresistance in OC^[176]^. Upregulated AR promotes paclitaxel resistance by complexing with aryl hydrocarbon receptor (Ahr), binding to an alternative ARE on ABCG2 promoter, increasing expression in taxol-resistant serous EOC^[177]^. Histone H1-0 knockdown decreases AR, sensitizing OC. PI3K/Akt overexpression elevates H1.0, which activates the GCN5, a histone acetyltransferase that acetylates the H3 histone, leading to AR-mediated ABCB1, and ABCG2 expression, ultimately conferring paclitaxel resistance^[178]^. Toll-like receptor 4 (TLR4) overexpression in taxol resistance OC activates AR, forming a TLR4/AR axis linked to taxol resistance^[179]^. Further study shows that AR is regulated by the TLR4/IL-6/interferon regulatory factor 1 (IRF1) signaling axis in OC^[180]^. NGF1-B and NOR1 from family 4, with class IV features, are associated with cisplatin chemoresistance. NR4A1 downregulation is observed in cisplatin-resistant OC^[181]^. Cisplatin-induced cytoplasmic translocation of NR4A1 is lower in resistant cell lines, where it regulates apoptosis through un N-terminal kinase (JNK) activation, Akt inhibition, and interaction with Bcl-2^[182]^. NR4A2 has also been reported to confer 5-fluorouracil and oxaliplatin resistance in gastric and colorectal cancer^[183,184]^. The NR4A3 has high expression in cisplatin resistance cell lines SKOV3 and A2780^[185]^.

### The interplay between nuclear receptors and OCSCs

The interaction between nuclear receptors and OCSCs promotes OC relapse and numerous research studies have uncovered their correlation. PPARγ, family 1, and class II member, linked with OCSCs, drives M2 polarization of Raw264.7 cells, inhibiting NF-κB, and GW9662, a PPARγ antagonist, counters these effects on macrophages^[186]^. COUP-TFγ from family 2, with class I features, is linked to cisplatin resistance by promoting EOC stem cells and sustaining Notch3 signaling by enrichment of p300 and increasing p300 acetylated histone acetylation H3(H9, K27) at Notch3 promoter^[187]^. ERβ, especially isoform 1, from family 3, exhibiting class I features, shows increased presence in OCSCs, while LY500307, an ERβ agonist, reduces OCSC viability and downregulates SOX2, OCT4, and NANOG^[188]^. The ERRα from family 3, with class IV features, is linked to OCSCs. The miR-200 family regulates Snail through ERRα, and reducing miR-200a/b expression reverses EMT and stem cell characteristics in OC^[189]^. AR from family 3 with class I features facilitates growth in CSPC-rich OVTC PA1 cells, governing the self-renewal of stem cells. AR is more abundant in CD133+ cells, and its enrichment downregulates p53 and p16^[190]^. AR is upregulated in OCSC and androgen 5α-dihydrotestosterone (DHT) promotes OC stemness by enhancing NANOG expression^[191]^.

## THE ROLE OF NCRNAS IN CHEMORESISTANCE IN OC

**Figure 4:**
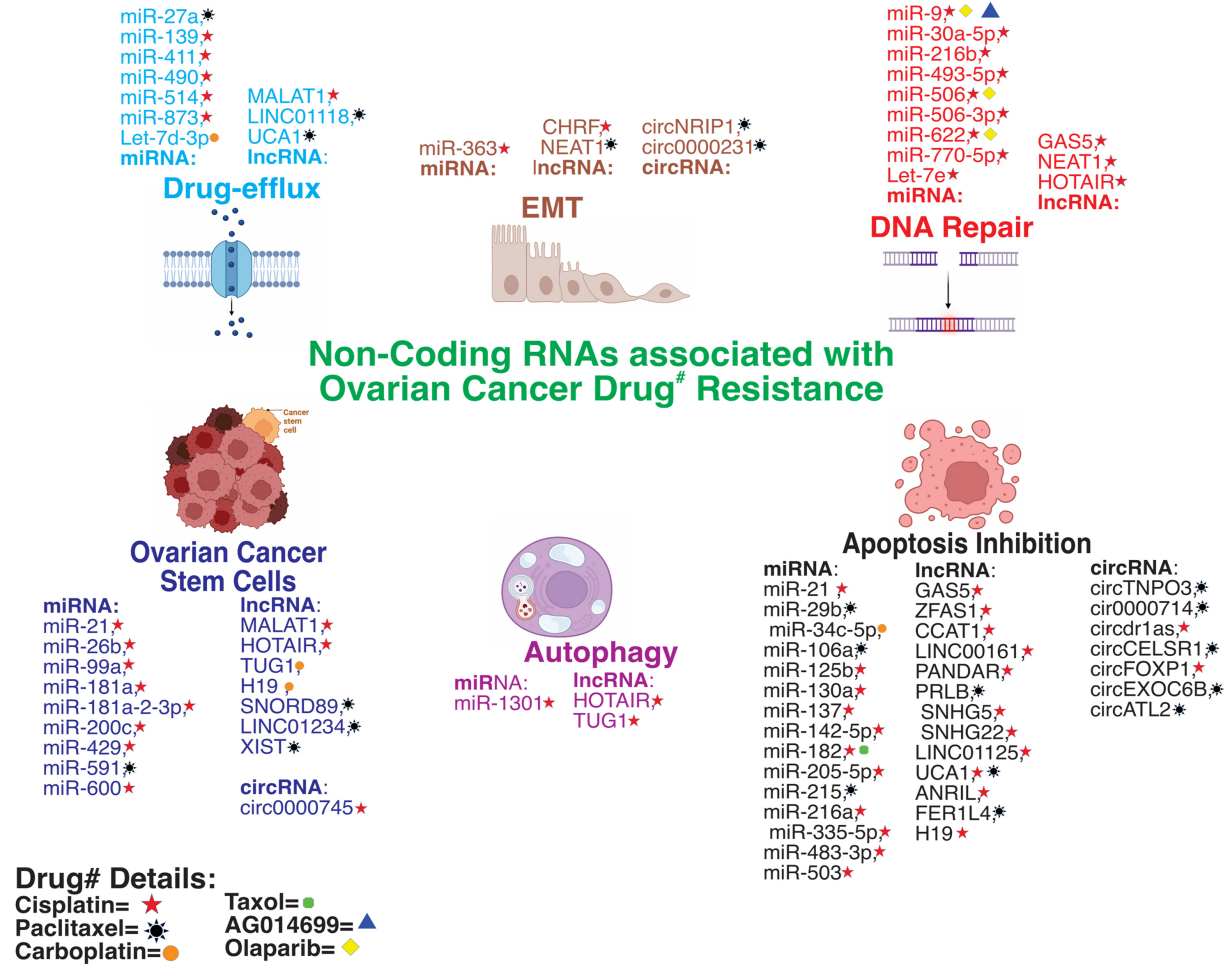
The well-defined mechanisms associated with various ncRNAs in OC drug^#^ resistance primarily involve autophagy, drug efflux, inhibiting cell apoptosis, DNA repair, EMT, and OCSCs. Created with BioRender.com and CorelDRAW. ncRNAs: Non-coding RNAs; OC: ovarian cancer; EMT: epithelial to mesenchymal transition; OCSCs: ovarian cancer stem cells.

ncRNAs serve as gene expression regulators across multiple biological processes, such as cell division, programmed cell death, cellular transport, EMT, OCSCs, and DNA mending^[192]^. Recent studies highlight ncRNAs as crucial regulators of chemoresistance in ovarian, breast, and lung cancers^[193-195]^. Understanding the identification and mechanisms of ncRNAs in gene expression regulation can aid in biomarker development for early detection. Additionally, targeting ncRNAs in chemotherapy can enhance cell death, ultimately leading to a higher 5-year survival rate. This review summarizes the roles of ncRNAs, particularly lncRNA, circRNA, and miRNA, in chemoresistance in OC [Table 6 and Figure 4].

**Table 6:** Summary of various non-coding RNAs (lnc-RNA, circular RNA and miRNA) involved in OC drug resistance, encompassing the details about specific drugs and their associated mechanism

**Name of non-coding RNAs**	**Differential expression patterns**	**Model system**	**Chemotherapy agent**	**Mode of mechanism**	**Target/pathway axis**	**Ref.**
**Long non-coding RNA**						
MALAT1	Up	A2780, OVCAR3, COC1	Cisplatin	Drug efflux	Notch1-ABCC1	[196]
		SKOV3	Cisplatin	OCSCs	YAP	[197]
GAS5	Down	HEY, A2780, HO8910, HO8910PM SKOV3, IOSE	Cisplatin	DNA repair; apoptosis inhibition	E2F4-PARP1-MAPK	[198]
ZFAS1	Up	SKOV3, Caov3, OVCAR3, A2780, COV644	Cisplatin	Apoptosis inhibition	miR-548e-CXCR4-let-7a/BCL-XL/S	[199]
CCAT1	Up	A2780, SKOV3	Cisplatin	Apoptosis inhibition	miR-454-survivin	[200]
LINC00161	Up	SKOV3	Cisplatin	Apoptosis inhibition	miR-128-MAPK1	[201]
LINC01118	Up	SKOV3, A2780, COC1	Paclitaxel	Drug efflux	miR-134-ABCC1	[202]
CHRF	Up	ES2	Cisplatin	EMT	miR-10b-STAT3	[203]
PANDAR	Up	SKOV3, HO-8910, HO8910PM, A2780	Cisplatin	Apoptosis inhibition	SFRS2-P53/P53-Ser15	[204]
PRLB	Up	CAOV3, SKOV3	Paclitaxel	Apoptosis inhibition	RSF1-NF-κB	[205]
SNHG5	Down	HeyA8, SKOV3	Paclitaxel	Apoptosis inhibition	miR-23a	[206]
SNHG22	Up	Hey, OAW28, COV362, OVCAR3, CAOV3, SKOV3, A2780	Cisplatin	Apoptosis inhibition	miR-2467-Gal-1	[207]
LINC01125	Down	SKOV3, A2780	Cisplatin	Apoptosis inhibition	miR-1972	[208]
NEAT1	Up	SKOV3, A2780	Cisplatin	DNA repair	miR-770-5p-PARP1	[209]
		SKOV3, HeyA8	Paclitaxel	EMT	miR-194-ZEB1	[210]
HOTAIR	Up	A2780, SKOV3, HEYC2, OV90, IOSE, IGROV, OVMUNA, OV90	Cisplatin	DNA repair	NF-κB	[211]
		SKOV3, A2780	Cisplatin	Autophagy	ATG7	[212]
		OVCAR3, CAOV3, OVCAR5, COV362, Kuramochi, HOSEs	Cisplatin	OCSCs	EZH2	[213]
		SKOV3, ES2, OVCAR3			miR-206-TBX3	[214]
UCA1	Up	SKOV3, HeyA8	Paclitaxel	Drug efflux	miR-129-ABCB1	[215]
		OAW42, OVCAR3	Cisplatin	Apoptosis inhibition	miR-27a-5p-UBE2N	[216]
		A2780, SKOV3, IOSE80	Cisplatin	Apoptosis inhibition	miR-143-FOSL2	[217]
		A2780, OAW42, OVCAR4, SKOV3, HeyA8, IOSE-386	Paclitaxel	Apoptosis inhibition	miR-654-5p-SIK2	[218]
ANRIL	Up	HOSEPiCs, SKOV3	Cisplatin	Apoptosis inhibition	let-7a-HMGA2	[219]
TUG1	Up	IOSE80, IOSE386 SKOV3, A2780	Paclitaxel	Autophagy	miR-29b-3p	[220]
		IOSE80, A2780, SKOV3, HO8910	-	OCSCs	186-5p-ZEB1	[221]
FER1L4	Down	IOSE80, HOSEpiC, OVCAR3, Caov3, SKOV3	Paclitaxel	Apoptosis inhibition	MAPK	[222]
H19	Up	A2780	Cisplatin	Apoptosis inhibition	EZH2-p21/PTEN	[223]
		SKOV3	Carboplatin	OCSCs	miR-29b-3p-STAT3	[224]
SNORD89	Up	HOSEpiC, OVCAR3, CAOV3	-	OCSCs	Notch1-c-Myc	[225]
LINC01234	Up	SKOV3, CAOV3, HO8910, A2780, IOSE80	-	OCSCs	miRNA-27b-5p-SIRT5	[226]
XIST	Down	SKOV3, ES2, TOV21G, RMG1	Paclitaxel	OCSCs	miR-93-5p-KMT2C	[227]
**Circular RNA**						
circTNPO3	Up	SKOV3, HeyA8, IOSE80	Paclitaxel	Apoptosis inhibition	miR-1299-NEK2	[228]
circNRIP1	Up	HOEC, A2780, SKOV3	Paclitaxel	EMT	miR-211-5p-HOXC8	[229]
circ0000714	Up	SKOV3, A2780	Paclitaxel	Apoptosis inhibition	miR-370-3p-RAB17	[230]
circdr1as	Down	IOSE80, A2780, SKOV3	Cisplatin	Apoptosis inhibition	miR-1270-SCAI	[231]
circCELSR1	Up	IOSE80, SKOV3, HeyA8	Paclitaxel	Apoptosis inhibition	miR-1252-FOXR2	[232]
circFOXP1	Up	COC1, OVCAR3, SKOV3, IOSE80	Cisplatin	Apoptosis inhibition	miR-22-CEBPG, circFOXP1-miR-150-3p-FMNL3	[233]
circEXOC6B	Down	IOSE80, A2780, SKOV3	Paclitaxel	Apoptosis inhibition	miR-376c-3p-FOXO3	[234]
circ0000231	Up	SKOV3	Paclitaxel	EMT	miR-140-RAP1B	[235]
circATL2	Up	IOSE80, HEYA8, SKOV3 Cells	Paclitaxel	Apoptosis inhibition	miR-506-3p-NFIB	[236]
circ0000745	Up	IOSE80, CoC1, ES2, SW626, SKOV3	-	OCSCs	miRNA-3187-3p-ERBB4/PI3K/AKT	[237]
**miRNA**						
miR-9	Down	CaOV3, SKOV3, OV2008, A2780	Cisplatin; AG014699	DNA repair	BRAC1	[238]
miR-21	Up	SKOV3, A2780	Cisplatin	Apoptosis inhibition	PDCD4-cIAP2	[239]
		PA1	-	OCSCs	-	[240]
miR-26b	Down	SKOV3	-	OCSCs	PTEN	[241]
miR-27a	Down	A2780	Paclitaxel	Drug efflux	HIPK2/MDR1/P-gp	[242]
miR-29b	Down	ES2, AMOC2	Paclitaxel	Apoptosis inhibition	BAG3/miR-29b/MCL-1	[243]
miR-30a-5p	Down	Cisplatin resistance OC cell	Cisplatin	DNA repair	RIF1	[244]
miR-34c-5p	Down	OVS1, SKOV-I6	Carboplatin	Apoptosis inhibition	AREG-EGFR-ERK	[245]
miR-99a	Down	IOSE80, HO8910, SKOV3	-	OCSCs	YY1	[246]
miR-106a	Up	SKOV3	Paclitaxel	Apoptosis inhibition	caspase-7, BCL10	[247]
miR-591	Down	SKOV3	Paclitaxel	OCSCs	ZEB1	[247]
miR-125b	Up	OV2008	Cisplatin	Apoptosis inhibition	BAK1	[248]
miR-130a	Down	A2780	Cisplatin	Apoptosis inhibition	XIAP	[249]
miR-137	Down	SKOV3, A2780	Cisplatin	Apoptosis inhibition	XIAP	[250]
miR-139	Down	CAOV3, SNU119	Cisplatin	Drug efflux	ATP7A/B	[251]
miR-142-5p	Down	OVCAR3, SKOV3	Cisplatin	Apoptosis inhibition	XIAP, BIRC3, BCL2, BCL2L2, MCL1	[252]
miR-181a	Up	OV81.2-CP10, OV236, OCI-P5X, HEYA8	Cisplatin	OCSCs	SFRP4	[253]
miR-181a	Down	OV90, SKOV3	Cisplatin; carboplatin	OCSC	CD24-miR-181a-MET	[254]
miR-181a-2-3p	Down	OC tumor clinical tissues	-	OCSCs	EGR1	[255]
miR-182	Up	T29, T80, HEY, OVCAR3, SKOV3, OV2008, 3AO, A2780, HO8910	Cisplatin; taxol	Apoptosis inhibition	PDCD4	[256]
miR-200c	Down	Caov3, OVCAR5, OVCAR8	-	OCSCs	Gab2	[257]
miR-205-5p	Up	OV2008	Cisplatin	Apoptosis inhibition	PTEN/AKT	[258]
miR-215	Down	OVCAR3, CAOV3, SKOV3, HEY	Paclitaxel	Apoptosis inhibition	XIAP	[259]
miR-216a	Up	SKOV3, OVCA433	Cisplatin	Apoptosis inhibition	PTEN	[260]
miR-216b	Down	SKOV3	Cisplatin	DNA Repair	PARP1	[261]
miR-335-5p	Down	A2780	Cisplatin	Apoptosis inhibition	BCL2L2	[262]
miR-363	Down	OV2008, A2780s	Cisplatin	EMT	Snai1	[263]
miR-411	Down	SKOV3, OVCAR3	Cisplatin	Drug efflux	ABCG2	[264]
miR-429	Down	SKOV3	Cisplatin	OCSCs	ZEB1	[265]
miR-483-3p	Up	IGROV1	Cisplatin	Apoptosis inhibition	PKC-alpha	[266]
miR-490-3p	Down	SKOV3, OVCAR3	Cisplatin	Drug efflux	ABCC2	[267]
miR-493-5p	Up	VU423, OVSAHO, kuramochi	Cisplatin	DNA repair	BRAC2	[268]
miR-503	Down	SKOV3	Cisplatin	Apoptosis inhibition	PI3K p85	[269]
miR-506	Down	HeyA8, OVCA433, SKOV3	Cisplatin; olaparib	DNA repair	RAD51	[270]
miR-506-3p	Down	SKOV3, CAOV3, OAW42, OV90	Cisplatin	DNA repair	RAD17	[271]
miR-514	Down	SKOV3, OVCA433	Cisplatin	Drug efflux	ABCA1, ABCA10, ABCF2	[272]
miR-600	Up	HO8910, A2780	-	OCSCs	KLF9	[273]
miR-622	Up	UWB1.289	Cisplatin; olaparib	DNA repair	Ku complex	[274]
miR-770-5p	Down	A2780S, OV2008	Cisplatin	DNA repair	ERCC1	[275]
miR-873	Down	OVCAR3, A2780	Cisplatin	Drug efflux	ABCB1	[276]
miR-1301	Down	SKOV3	Cisplatin	Autophagy	E-cadherin, N-cadherin, ATG5, beclin1	[277]
Let-7d-3p	Up	SKOV3	Carboplatin	Drug efflux	ABC transporters, HIF-1, RAS, ErbB	[278]
Let-7e	Down	A2780, HO8910, ES2, CAOV3, SKOV3, OV2008	Cisplatin	DNA repair	BRAC1, Rad51	[279]
		A2780, SKOV3, Caov3			PARP1	[280]

OC: Ovarian cancer; MALAT1: metastasis associated lung adenocarcinoma transcript 1; Notch1: neurogenic locus notch homolog protein 1; YAP: yes-associated protein; GAS5: growth arrest-specific transcript 5; E2F4: E2F transcription factor 4; PARP1: poly (ADP-ribose) polymerase 1; MAPK: mitogen-activated protein kinase; ZFAS1: zinc finger antisense 1; CXCR4: C-X-C motif chemokine receptor 4; CCAT1: colon cancer associated transcript-1; LINC: long intergenic non-protein coding RNA; CHRF: cardiac hypertrophy related factor; EMT: epithelial to mesenchymal transition; STAT3: signal transducer and activator of transcription 3; SFRS2: serine/arginine-rich splicing factor 2; RSF1: remodeling and spacing factor 1; NF-κB: nuclear factor kappa-light-chain-enhancer of activated B cells; NEAT1: nuclear enriched abundant transcript 1; ZEB1: zinc-finger E-box-binding homeobox 1; HOTAIR: HOX transcript antisense RNA; ATG7: autophagy related 7; UCA1: urothelial cancer associated 1; ABCB1: ATP-binding cassette subfamily B member 1; UBE2N: ubiquitin-conjugating enzyme E2 N; FOSL2: FOS like 2; SIK2: salt inducible kinase 2; ANRIL: antisense noncoding RNA in the INK4 locus; HMGA2: high mobility group A 2; TUG1: taurine-upregulated gene 1; FER1L4: fer-1 like family member 4; PTEN: phosphatase and tensin homolog deleted on chromosome 10; SIRT5: silent information regulator 5; NEK2: NIMA-related kinase 2; CircNRIP1: circular nuclear receptor interacting protein 1; HOXC8: homeobox protein; SCAI: suppressor of cancer cell invasion; FOXR2: forkhead box R 2; CEBPG: CCAAT enhancer binding protein gamma; FMNL3: formin-like 3; FOXO3: forkhead box O 3; PI3K: phosphatidylinositol 3 kinase; AKT: Ak strain transforming; BRAC1: breast cancer gene 1; PDCD4: programmed cell death 4; HIPK2: homeodomain interacting protein kinase 2; MDR1: multidrug resistance protein 1; P-gp: P-glycoprotein 1; BAG3: BAG cochaperone 3; MCL-1: myeloid cell leukemia sequence 1; AREG: amphiregulin; EGFR: epidermal growth factor receptor; ERK: extracellular-signal-regulated kinase; BCL10: B-cell lymphoma/leukemia 10; BAK1: BCL2-antagonist-killer 1; XIAP: X-linked inhibitor of apoptosis protein; BIRC3: baculoviral IAP repeat-containing protein 3; BCL2: B-cell lymphoma 2; BCL2L2: BCL2 like 2; SFRP4: secreted frizzled-related protein 4; MET: mesenchymal epithelial transition; EGR1: early growth response factor 1; Snai1: snail family transcriptional repressor 1; ABCG2: ATP-binding cassette subfamily G member 2; PKC-alpha: protein kinase C alpha; ABCC2: ATP-binding cassette subfamily C member 2; BRAC2: breast cancer gene 1; ABCA1: ATP-binding cassette subfamily A member 1; ABCA10: ATP-binding cassette subfamily A member 10; ABCF2: ATP-binding cassette sub-family F member 2; ERCC1: excision repair cross-complementation group 1; E-cadherin: epithelial cadherin; N-cadherin: neural cadherin; ATG5: autophagy related 5; HIF-1: hypoxia-inducible factor 1; RAS: rat sarcoma; ErbB: erythroblastic leukemia viral oncogene homolog.

## CONCLUSION

Chemoresistance poses a significant challenge in cancer treatment, contributing to elevated mortality rates among OC patients. Despite limited understanding, this review delineates four pivotal factors, namely transport systems, DNA repair, apoptosis, and OCSCs, along with the regulatory roles of ncRNA and nuclear receptors in conferring chemoresistance in OC. The involvement of specific transporters, including ABCB1, ABCC1, ABCC4, ABCC10, ABCG2, P-type ATPases ATP7A and ATP7B, and SLC transporters CTR1 and CTR2, are found in the development of chemoresistance. Additionally, proteins such as Bcl-2, Bcl-XL, MCL-1, Bax, Bim, XIAP, survivin, and cIAP2, along with ubiquitination proteins (HOIP, ITCH. CRL4 and UBR5), glycosylation, galectins (galectin-1, and -3), and epigenetic regulation (DNA methyltransferase, HDAC, and hMOF) contribute to chemoresistance in OC. Disruption of extrinsic pathways through TRAILR2 and FLIP further enhances chemoresistance. DNA repair mechanisms play a crucial role, with components like HR (BRCA1/2, RAD51, and its paralogs RAD51C, and RAD51D, RAD52, MRE11), NHEJ (RIF1), NER (ERCC1, XPF, RPA1), MMR (hMLH1, hMSH2), and BER (APE1, and XRCC1) identified as factors responsible for chemoresistance development. OCSCs are recognized as key contributors to tumor relapse, identified through various markers like CD133, CD24, CD44, CD117, ALDH1, SOX2, OCT4, NANOG, and EpCAM. ncRNA, as revealed in recent studies, exerts roles in tumor relapse by influencing drug efflux, apoptosis inhibition, DNA repair, EMT, autophagy, and OCSCs. Moreover, nuclear receptors (PPARγ, LXRα/β, PXR, CAR, COUP-TFγ, ERα, ERβ, ERRα AR, NGF1-B, NOR1) have emerged as significant contributors to chemoresistance in OC, modulating apoptosis, drug efflux, and OCSCs. The current treatment approach for the heterogeneous nature of OC lacks a multifactorial perspective. ncRNA and nuclear receptors, given their regulatory influence on multiple gene expressions, hold promise for targeted therapies. Exploring OCSCs further and understanding their role in promoting tumor relapse can guide effective interventions. Ongoing research utilizing advanced technology is expected to uncover additional resistance mechanisms, paving the way for tailored or combination therapies that enhance the survival of OC patients.

## DECLARATIONS

### Availability of data and materials

The corresponding author may be contacted for any data inquiries.

### Copyright

© The Author(s) 2024.
